# MRI–Ultrasound Fused Approach for Prostate Biopsy—How It Is Performed

**DOI:** 10.3390/cancers16071424

**Published:** 2024-04-07

**Authors:** Jacob Lang, Timothy Dale McClure, Daniel J. A. Margolis

**Affiliations:** 1 Department of Urology, Weill Cornell Medicine, New York, NY 10068, USA; 2 Department of Radiology, Weill Cornell Medicine, New York, NY 10068, USA

**Keywords:** prostate, cancer, MRI, biopsy

## Abstract

**Simple Summary:**

Prostate cancer screening has traditionally been accomplished by a blood test for prostate serum antigen (PSA) followed by biopsy. MRI is very accurate at finding cancer, but ultrasound provides real-time guidance for biopsy. Using magnetic resonance imaging (MRI) to guide the biopsy under ultrasound improves detection and, in the case that only non-aggressive cancer is found, confidence in avoiding treating cancers that are unlikely to be deadly. Different approaches can be used with many different MRI–ultrasound fusion techniques, including “cognitive” fusion using only the practitioner’s reading of the scans, but also software fusion using mechanical or even pure software-aided matching of the information from MRI and ultrasound.

**Abstract:**

The use of MRI–ultrasound image fusion targeted biopsy of the prostate in the face of an elevated serum PSA is now recommended by multiple societies, and results in improved detection of clinically significant cancer and, potentially, decreased detection of indolent disease. This combines the excellent sensitivity of MRI for clinically significant prostate cancer and the real-time biopsy guidance and confirmation of ultrasound. Both transperineal and transrectal approaches can be implemented using cognitive fusion, mechanical fusion with an articulated arm and electromagnetic registration, or pure software registration. The performance has been shown comparable to in-bore MRI biopsy performance. However, a number of factors influence the performance of this technique, including the quality and interpretation of the MRI, the approach used for biopsy, and experience of the practitioner, with most studies showing comparable performance of MRI–ultrasound fusion to in-bore targeted biopsy. Future improvements including artificial intelligence promise to refine the performance of all approaches.

## 1. Introduction

Prostate cancer (PCa) is the most common cancer diagnosis and the second leading cause of cancer-related death in men in the United States (US), with similar prevalence and mortality in the European Union (EU) [1,2]. This prevalence is largely due to increased detection due to advances in PCa screening practices. However, many of these cancers are indolent and of low metastatic potential, thus considered clinically insignificant PCa (cisPCa) [3].

Systematic transrectal ultrasound (TRUS)-guided biopsy was previously the standard of care for diagnosing PCa and is associated with high false negative rates and underdiagnosis of clinically significant PCa (csPCa) [4,5]. The advent of multiparametric magnetic resonance imaging (mpMRI) of the prostate and its incorporation into targeting lesions for prostate biopsy has considerably changed prostate cancer evaluation and diagnosis through improved detection of csPCa and a reduction in detection of clinically insignificant cancers [6,7,8,9,10]. mpMRI-guided biopsy has gained widespread use due to improved detection and accuracy of csPCa over systematic biopsy alone and is now recommended in major national and international PCa guidelines [4,11].

This review aims to provide an overview of how mpMRI-guided biopsy is performed, including mpMRI and the identification of target lesions, current indications, and an overview of mpMRI-guided techniques and their comparative effectiveness, limitations, and future directions.

## 2. mpMRI and Identification of Lesions

Utilization of mpMRI for PCa evaluation has substantially increased over the past decade and plays an integral role in the prostate biopsy decision-making and targeting process [12,13]. Current mpMRI technology utilizes four sequences, T1- and T2-weighted images, diffusion-weighted images (DWI), and dynamic contrast-enhanced imaging (DCEI), to depict detailed anatomy and identify suspicious lesions. T2-weighted and DWI sequences, in combination, are the most prominent in identifying suspicious lesions [14].

To standardize radiologic evaluation of lesions, the Prostate Imaging Reporting and Data System (PI-RADS) scoring system was established in 2012 by the European Society of Urogenital Radiology (ESUR). PI-RADS utilizes standardized criteria to evaluate suspicious lesions on mpMRI, resulting in an assigned score from 1 to 5 (very low to very high suspicion). It has since been updated, first with v2.0 in 2014, which established a dominant sequence for evaluation with DWI for the peripheral zone and T2-weighted imaging for the transition zone, decreased the importance of DCEI in evaluation, and removed magnetic resonance spectroscopic imaging (MRSI), resulting in improved sensitivity of detecting csPCa when compared to v1 in head-to-head trials, but no difference in specificity [15].

In 2019, PI-RADS v2.1 further refined the scoring system via changes including additional T2-weighted images, a clarified role of *b* value interpretation for DWI apparent diffusion coefficient (ADC) map use, and revisions in criteria for DWI scores 2 and 3 as well as an evaluation of lesions in the central zone and anterior fibromuscular stroma [16,17]. Current recommendations for mpMRI for fusion biopsy include use of a 1.5 Tesla (T) or higher field strength scanner (3 T when available and feasible) with an endorectal coil when available. They should at minimum include T2-weighted imaging, DWI with ADC mapping with at least one high *b* value ≥ 1400 s/mm^2^, one intermediate *b* value ~800 s/mm^2^, and one low *b* value ≤ 100 s/mm^2^ to ensure optimal contrast, and DCEI [16,17]. With these changes, a 2021 systematic review and meta-analysis of PI-RADS v2.1, the pooled sensitivity and specificity for detecting csPCa were 87% (95% CI, 82–91%) and 74% (63–82%), respectively, with no statistically significant difference from v2.0 [18].

## 3. Current Indications

Most current national and international guidelines define an abnormal mpMRI as PI-RADS ≥ 3, with caveats for significantly abnormal mpMRI to PI-RADS 4 or 5 based on local expertise. The Prostate MR Imaging Study (PROMIS) was a multicenter, paired-cohort study comparing mpMRI via template mapping biopsy to TRUS biopsy. The investigators found a significantly higher sensitivity for mpMRI, 93% (95% CI 88–96%) versus 48% (95% CI 42–55, *p* < 0.0001), as well as a high negative predictive value of 89% (95% CI 83–94%) for PI-RADS lesions < 3, and thus the ability to avoid biopsy in these cases [8]. A 2020 systematic review and meta-analysis of prospective studies showed pooled detection rates of csPCa sequentially increased for each PI-RADS v2 category [4% (95% CI 2–8) for 1–2, 17% (95% CI 13–21) for 3, 46% (95% CI 38–55) for 4, and 75% (95% CI 73–78) for 5] [19]. Because of the equivocal nature of PI-RADS 3 lesions and lower demonstrated yield of csPCa in these lesions, clinical correlation with other clinical factors, including PSA density, age, biopsy-naïve status, and prior negative biopsy to assess risk for csPCa, is recommended [20,21].

mpMRI-guided biopsy techniques are currently recommended or provided as an option by major guideline societies, including the American Urological Association (AUA), Society of Urologic Oncology (SUO), National Comprehensive Cancer Network (NCCN), and European Association of Urology (EAU), predominantly for biopsy-naïve men and men with prior negative biopsy [22,23,24]. AUA guidelines provide a conditional recommendation that clinicians may use MRI prior to initial biopsy to increase detection of csPCa, and moderately recommend targeted biopsy in biopsy-naïve patients with a suspicious lesion on MRI. In patients with an indication for repeat biopsy without prior prostate mpMRI, obtaining mpMRI is currently strongly recommended with subsequent targeted biopsy if abnormal, with an optional additional systematic biopsy. In EAU guidelines, mpMRI with subsequent biopsy if positive is strongly recommended in biopsy-naïve patients. Additionally, patients with prior negative biopsies with an indication for repeat biopsy are recommended to undergo mpMRI with targeted biopsy alone if abnormal. In the NCCN Prostate Cancer Early Detection guidelines, mpMRI-guided techniques are strongly recommended to be employed routinely. A list of guidelines and recommendations from major US and EU associations is displayed in Table 1.

## 4. Overview of mpMRI-Guided Biopsy Techniques

After an mpMRI is obtained and read using the PI-RADS v2.1 scoring system, identified regions of interest must be segmented for targeting. T2-weighted imaging is most commonly utilized for fusion due to its spatial resolution and decreased vulnerability to susceptibility artifacts and geometric distortion compared to DWI [16,25,26].

Three predominant techniques for mpMRI targeting are commonly utilized: cognitive fusion, mpMRI-TRUS image fusion, and MRI in-bore/in-gantry techniques. Initially, lesions were targeted via cognitive fusion, i.e., visual estimation of lesion location on TRUS based on mpMRI images. The subsequent development of image fusion technologies, as well as in-scanner, MRI-guided techniques, has allowed for more precise identification of lesions. mpMRI–TRUS image fusion biopsy utilizes mpMRI images and TRUS to identify and target lesions via the transrectal (TR) or transperineal (TP) approach. MRI direct in-bore or in-gantry targeting technologies allow for in-scanner identification and targeting of lesions. A list of some available systems is provided in Table 2.

### 4.1. Cognitive Fusion

Cognitive fusion was the original method of fusion biopsy in which the operator reviews mpMRI and visually registers and targets the lesion via anatomic positioning on TRUS. Advantages of cognitive fusion include its relatively low cost, as it requires no additional software or equipment, low relative procedural time, as there is no need for software segmentation pre- or intraprocedurally, and ease of concurrent systematic biopsy [27]. It can be performed via TR or TP approach. Cognitive fusion biopsy is limited by operator dependence, which can be negatively affected by inexperience with either mpMRI or TRUS-guided biopsy [27].

### 4.2. mpMRI–TRUS Fusion

mpMRI–TRUS image fusion techniques utilize software to register mpMRI target lesion(s) to a corresponding anatomic location on TRUS and provides a guide for the biopsy needle. mpMRI images are segmented prior to or during the biopsy procedure. mpMRI and TRUS images are then registered by one of two types of registration, rigid or elastic. Rigid registration aligns mpMRI and TRUS images without altering their shapes and only accounts for rotational or translational differences, i.e., the operator must manually correct for distortions during the procedure. Elastic registration adjusts for intraprocedural changes in US images, such as alterations due to mass effect by the TRUS probe or prostate deformation from adjacent structures. Despite the perceived advantages of elastic registration, a 2016 meta-analysis comparing the two methods demonstrated no difference in the detection of PCa [28]. Continuous tracking of the real-time position of the US probe and needle intra-procedurally is performed via several methods, including electromagnetic tracking (e.g., UroNav, Philips Healthcare), position-encoded sensors in smart robotic arms (e.g., Artemis, Eigen), and image-based software tracking (e.g., Trinity, Koelis). Additionally, targeting can be prospective, in which the software displays the biopsy needle tract before biopsy, or retrospective, in which a scan is taken after the biopsy needle is deployed to confirm positioning in the target lesion.

Similar to cognitive fusion, image fusion allows for ease of concurrent systematic biopsy and is widely available. Disadvantages in comparison to cognitive fusion include increased cost due to required software/hardware and increased relative procedural time due to image registration. This technique can also be performed via TR or TP approach. One single-center study demonstrated that the learning curve in terms of timing, csPCa detection, and pain for operators for mpMRI targeted biopsy is a minimum of 50 cases [29]. Another study demonstrated improving csPCa detection rates with experience but showed no difference in detection rates by operator seniority [30].

### 4.3. Direct In-Bore/In-Gantry

MRI direct in-bore, or in-gantry, targeting is performed in the MRI scanner (e.g., DynaTRIM, Philips Healthcare). The patient is positioned head first and prone, and a needle guide is rectally or transperineally placed. The operator then obtains axial T2-weighted imaging and DWI sequences to identify the ROI. A fast, steady-state free precession image is then obtained, and the biopsy needle sequentially advanced with its position confirmed through repeated scans [31]. It can be performed via TR or TP approach. This method is the least commonly used due to its increased costs and procedural time (30–60 min), compared to both cognitive and image fusion techniques [3,31,32,33].

## 5. Comparative Effectiveness

### 5.1. mpMRI-Guided versus Systematic TRUS Biopsy

As noted previously, all three mpMRI-guided biopsy techniques have demonstrated advantages in diagnosing csPCa and reducing the diagnosis of cisPCa over systematic biopsy. The 2018 PRECISION trial was a multicenter, randomized, controlled noninferiority trial comparing mpMRI-targeted biopsy versus systematic TRUS-guided biopsy in 500 biopsy-naïve men. In this study, mpMRI-targeted biopsy had a significantly higher rate of detection for csPCa, defined as Gleason ≥ 3 + 4, at 38% versus 26% for TRUS biopsy (*p* < 005), and a significantly lower rate of cisPCa (9% versus 22%, *p* < 0.001) [7].

A 2019 Cochrane Systematic Review was consistent with these findings; in the mpMRI pathway with targeted biopsy in men with prior negative biopsy or who were biopsy-naïve, there was a pooled detection ratio (mpMRI pathway detection rate:systematic TRUS biopsy pathway rate) of 1.12 (95% CI, 1.02–1.23, 25 studies) for csPCa. When evaluated separately, the detection ratio was higher for men with prior negative biopsy than biopsy-naïve men, with the latter just below statistical significance in its meta-analysis. They concluded the MRI pathway has the most favorable diagnostic accuracy in detection of csPCA, while also decreasing the detection of cisPCa. [6]

Regarding cognitive fusion biopsy alone, several studies have demonstrated improved detection of csPCa and greater concordance with final histopathology compared to systematic biopsy [34,35,36,37,38]. Another study showed TP cognitive biopsy had similar csPCa detection rates to TP template biopsy, although the detection rate of clinically insignificant cancer was lower [39].

For in-bore techniques, a 2018 large, prospective, multicenter head-to-head study of in-bore biopsy versus systematic TRUS biopsy in 626 men found similar rates of detection of csPCa, but a significantly lower rate of detection for cisPCa for the in-bore technique, reducing the number of men requiring biopsy by 49% [10].

A more recent 2022 meta-analysis found a significantly higher csPCa detection rate for all mpMRI-guided biopsy techniques versus TRUS-guided biopsy with a pooled relative cancer detection rate of 1.24 (95% CI, 1.03–1.50, *p* = 0.02), as well as a significantly lower cisPCa yield with a pooled relative yield of 0.58 (95% CI, 0.46–0.74) compared to TRUS-guided biopsy, in line with other previous meta-analyses [9,38,40].

Given the data demonstrating the superior performance of mpMRI-guided biopsy in detecting csPCa, there is ongoing debate regarding the necessity of concurrent systematic TRUS biopsy. However, several studies have shown an improved diagnostic yield of csPCA, with reported rates as much as 20% higher when systematic TRUS biopsy is performed in addition to mpMRI-guided biopsy [10,41,42,43,44,45,46,47,48]. A prospective National Cancer Institute (NCI) study of 2103 patients who underwent both mpMRI-targeted and systematic biopsy showed improved csPCa detection rates for mpMRI-targeted biopsy. However, when analyzing MRI-targeting alone, 8.8% of csPCa (grade group ≥ 3 in this study) were misclassified, and 8.7% upgraded on histopathological analysis at radical prostatectomy in comparison to combined biopsy (3.5%) [45]. The multicenter, paired diagnostic MRI-FIRST study of 251 biopsy-naïve men who underwent MRI-targeted TR biopsy and systematic TRUS biopsy also demonstrated combined improvement in detecting csPCa [45]. Similarly, in the PAIREDCAP, a paired diagnostic trial of 248 biopsy-naïve men who underwent systematic biopsy plus cognitive and image fusion biopsy, the combined approach yielded an additional 11% of csPCa [43]. Furthermore, inaccurate co-registration and targeting have been shown to introduce error while utilizing fusion technology [49,50]. Thus, the combination of mpMRI-targeted plus systematic TRUS biopsy remains included in international guideline statements with differing strengths of recommendation and consideration (Table 1).

### 5.2. Comparison across mpMRI-Guided Biopsy Techniques

Based on current evidence, there is no consensus for the best mpMRI-guided technique for prostate biopsy. The 2018 multicenter, randomized, controlled FUTURE Trial prospectively compared all three techniques in 665 patients. The investigators found no significant difference in PCa or csPCa detection rates across cognitive fusion, image fusion, or in-bore techniques. The study was noted to be limited by a low rate of PIRADS ≥ 3 lesions on mpMRI, potentially underpowering their findings. [51]

Image fusion biopsy techniques have demonstrated improved accuracy over cognitive fusion in several single-institution studies [52,53,54]. In recent meta-analyses and numerous single institutional studies, however, there have been no demonstrated significant differences between cognitive biopsy and image fusion techniques in terms of csPCa detection rates [36,38,55,56,57,58,59]. Despite similar detection rates, image fusion biopsy has been shown to have improved detection rates of anterior and transition zone lesions, while cognitive fusion has been shown to have improved detection of lesions at the base, suggesting a potential complementary effect of these techniques [27,36,56,57].

Micro-ultrasound technology is a recent advancement in US imaging that utilizes high-frequency imaging at 29 MHz, allowing for visualization of MRI lesions in real time. Prospective trials have demonstrated improved sensitivity over mpMRI and improved detection of csPCa for TR cognitive biopsy with micro-ultrasound compared to transperineal image fusion biopsy [60]. Furthermore, micro-ultrasound technology is low cost and can be performed in a single session, necessitating a larger-scale study of its potential in improving cognitive biopsy [61,62].

Regarding in-bore or in-gantry biopsy, several retrospective studies and one small prospective study have demonstrated improved detection of overall PCa and csPCa than cognitive or image fusion methods [51,63,64,65]. This is in contrast to large, randomized studies, which to date have demonstrated no significant difference in csPCa detection [51,66].

Perhaps most notably, updated systematic reviews and meta-analyses have consistently demonstrated similar detection rates across all three techniques across indications [38,40]. Thus, no mpMRI-guided technique has received a preferential recommendation in national and international guidelines (Table 1).

### 5.3. TP vs. TR Approach

All three mpMRI-guided biopsy techniques can be performed via TP and TR approaches. The TR approach has historically been advantageous in terms of cost and time but disadvantaged by increased risk of sepsis up to 7%. The TP approach offers reduced risk of infection, as well as improved anterior and apical sampling, but is limited by the requirement of general anesthesia [67]. The introduction of the freehand technique (e.g., PrecisionPoint Transperineal Access System, Perineologic) paired with effective local anesthesia has decreased the procedural time allowed for office-based practice, thus rapidly increasing adaptation of the TP approach [68,69].

CsPCa detection rates between the two techniques have been similar, with a few caveats. A 2022 systematic review and meta-analysis of 11 studies demonstrated no significant difference in the overall detection of csPCa; however, TP demonstrated significantly higher detection rates in apical (OR 1.86; 95% CI, 1.14–3.03; *p* = 0.01) and anterior (OR 2.17, 95% CI 1.46–3.22; *p* < 0.001) lesions, as well as significantly higher rates of detection for PI-RADS 4 lesions, with no difference for PI-RADS 3 and 5 lesions. This review was, however, limited by the retrospective design of most of the included studies [70]. Recently, the 2023 multicenter, randomized, controlled PREVENT Trial compared outcomes for mpMRI-guided and systematic biopsy via TP approach without antibiotic prophylaxis versus TR with targeted prophylaxis in 658 patients. The rates of infectious complications were 0% and 1.4% for TP and TR, respectively, just outside of statistical significance (*p* = 0.059). CsPCa detection rates were similar, 53% for TP versus 50% for TR [71]. Further data on csPCa detection rates are expected in 2024; the TRANSLATE trial, a multicenter, randomized controlled trial comparing TP versus TR mpMRI-guided plus systematic biopsy in 1042 patients, remains in the accrual phase [72]

### 5.4. Case Presentation

**Figure 1:**
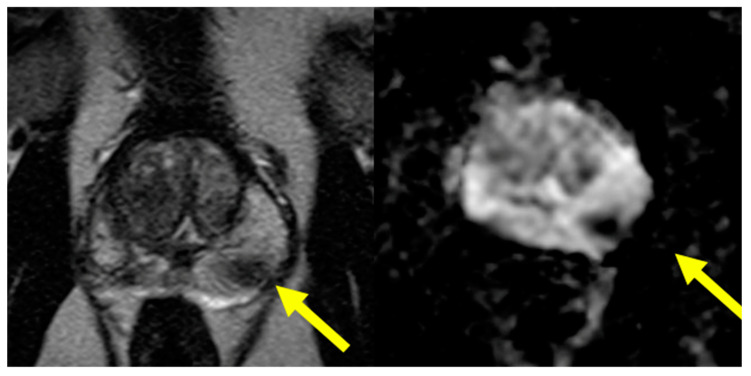
Axial T2-weighted (**left**) and axial ADC images (**right**) with arrow identifying the ROI.

The following is a case presentation and description of an mpMRI-guided biopsy via the TP approach performed at our institution. A 62-year-old male presented with a rise in prostate specific antigen (PSA) from baseline ~2 to ~7. The patient underwent 3.0 T mpMRI, which demonstrated a PI-RADS 4 lesion in the left posterior mid-gland that can be seen on T2-weighted (Figure 1, left) and ADC images (Figure 1, right).

After counseling on the risks and benefits of different biopsy options, the patient elected to undergo TP biopsy. The performing author (T.M.) at our institution predominantly utilizes mpMRI software fusion biopsy, specifically the UroNav system (Philips Healthcare) for TP biopsy [73]. This system was updated in 2018 to include a TP stepper with a grid for ease of TP biopsy targeting.

This software requires pre-biopsy segmentation of the prostate and targeted lesion via manual (most commonly) or automated methods. Our institution offers TP biopsy under local anesthesia or sedation per patient preference. As mentioned previously, UroNav works via electromagnetic tracking through a field generator and US probe manipulated via a robotic arm with multiple degrees of freedom. The UroNav cart and ultrasound machine is placed alongside the patient. The patient is given an enema prior to the procedure. The patient is placed in the dorsal lithotomy position. The scrotum is then elevated and secured superiorly. The field generator is positioned over the patient’s pelvis. The perineum is then prepped with chlorhexidine, sterilely draped, and 1% lidocaine is injected to provide local anesthesia. Antibiotics are then administered perioperatively. The US probe is covered with an endocavity balloon containing ultrasound gel. A TP stepper with a grid is then connected to the US probe for tracking. The US probe is then inserted into the rectum and the stepper aligned.

**Figure 2:**
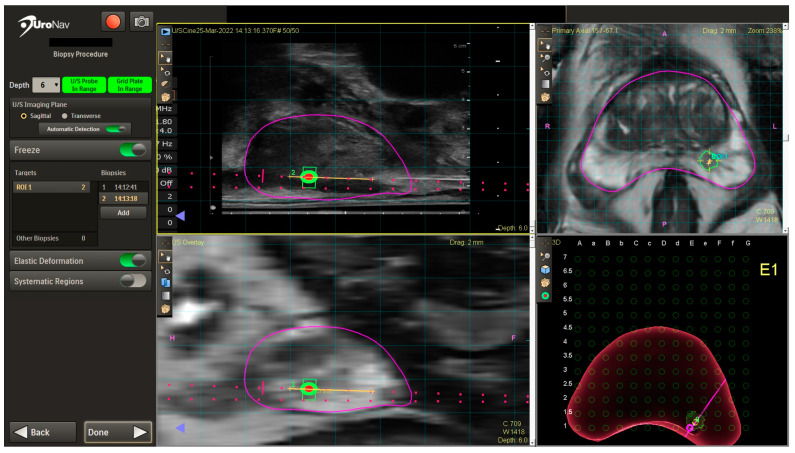
UroNav TP biopsy display images with ROI highlighted. The magenta outline defines the prostate. The orange line shows the biopsy trajectory. The green trapezoid is the radiologist-defined region of interest, whereas the green circle with orange center is the ideal “target”.

A TRUS sagittal sweep of the prostate is then performed to obtain TRUS images and dimensions, which are then fused with mpMRI images in real time on the UroNav system screen (Figure 2). After fusion, the UroNav display includes the identified ROI in the left posterior mid-gland with sagittal views (left upper and lower quadrants), axial view (right upper quadrant), and 3D rendered image with grid overlay for targeting (right lower quadrant). Biopsies are then obtained using grid holes corresponding to the location of the ROI. Cores are then taken from the ROI (four cores in this case). A systematic biopsy is then generally performed in biopsy-naïve patients. The UroNav system stores the biopsy needle trajectory for potential future use should the patient need another biopsy. At the conclusion of the procedure, the US probe is removed, perineum cleaned, and bacitracin applied. No post-procedure antibiotics or pain medications are prescribed at our institution.

At follow up, pathology from the ROI demonstrated prostatic adenocarcinoma, Grade Group 3 (Gleason score 4 + 3 = 7), involving 70%, 50%, and 30% (5 mm, 5 mm, 3.5 mm) of 3/4 cores. Gleason pattern 4 constituted 50–60% of the total tumor volume. At our institution TP biopsy is a collaboration with Urology and Interventional Radiology, with ~500 TP biopsies performed since 2018 and no reported cases of sepsis. Through this collaboration we have also begun to offer same day mpMRI, consultation, and potential biopsy.

### 5.5. Future Directions

Application of deep learning models (DLMs) to mpMRI lesion detection has shown promise in improving detection of csPCa. A 2024 study comparing a DLM integrating three individual mpMRI sequences using neural networks to clinical PI-RADS score in classification of csPCa and cisPCa demonstrated improved sensitivity, specificity, and accuracy over PI-RADS score [74]. A similar 2021 study of a texture-based DLM developed using T2-weighted and ADC images in comparison to PI-RADS classification also showed improved specificity, as well as overall area under the ROC curve (AUC), with greatest improvements in detection of peripheral zone and solitary tumor lesions on sub-analyses [75]. Incorporation of DLMs into clinical practice represents an emerging area of investigation to enhance classification of csPCa and cisPCa [76].

Micro-ultrasound offers the ability to detect lesions seen on mpMRI in real time at a low cost and demonstrates similar rates of detection for mpMRI-guided biopsy. It is not yet known whether use alone or in conjunction with mpMRI-guided methods is optimal for detection of csPCa. The ongoing three-armed randomized-controlled OPTIMUM trial comparing csPCa detection rates of micro-ultrasound alone versus mpMRI-US fusion biopsy versus mpMRI/micro-ultrasound with micro-ultrasound-based fusion device aims to address these questions [77,78].

## 6. Conclusions

mpMRI-guided prostate biopsy techniques have transformed the diagnosis, staging, and treatment of PCa through improved detection of csPCa and reduced diagnosis of cisPCa. This has led to recommended use in multiple national and international guidelines, predominantly for patients who are biopsy-naïve or with a prior negative biopsy. Cognitive fusion, image fusion, and in-bore/in-gantry techniques have similar rates of csPCa detection, and are all suitable targeting techniques that can be employed based on local circumstances. However, current evidence suggests combination with systematic biopsy results in the highest csPCa detection rates. TP and TR approaches to biopsy have similar csPCa detection rates. However, the TP approach is advantageous in the detection of csPCa in apical and anterior lesions, as well as in lower infectious risk and antibiotic stewardship. Lastly, the advent of DLMs for mpMRI classification and micro-ultrasound technology for real-time detection of lesions may further augment biopsy practice. Randomized controlled trials evaluating optimal mpMRI-guided biopsy techniques are ongoing.

## Figures and Tables

**Table 1:** Current guideline recommendations and indications for mpMRI-guided biopsy. “mpMRI” and “MRI” are used interchangeably, as both terminologies are used across different guidelines.

Society—*Guideline*	Statement(s)	Recommendation (Grade)
AUA/SUO— *Early Detection of Prostate Cancer*	Clinicians may use MRI prior to initial biopsy to increase the detection of GG2+	Conditional Recommendation; Evidence Level (Grade B)
	For biopsy-naïve patients who have a suspicious lesion on MRI, clinicians should perform targeted biopsies of the suspicious lesion and may also perform a systematic template biopsy.	Moderate Recommendation [targeted biopsies]/Conditional Recommendation [systematic template biopsy]; Evidence Level (Grade C)
	In patients undergoing repeat biopsy with no prior prostate MRI, clinicians should obtain a prostate MRI prior to biopsy.	Strong Recommendation; Evidence Level: Grade C)
	In patients undergoing repeat biopsy and who have a suspicious lesion on MRI, clinicians should perform targeted biopsies of the suspicious lesion and may also perform a systematic template biopsy.	Moderate Recommendation [targeted biopsies]/Conditional Recommendation [systematic template biopsy] Evidence Level: Grade C
	Clinicians may use software registration of MRI and ultrasound images during fusion biopsy, when available.	Expert Opinion
	Clinicians should obtain at least 2 needle biopsy cores per target in patients with suspicious prostate lesion(s) on MRI.	Moderate Recommendation; Evidence Level: Grade C
European Association of Urology/European Association of Nuclear Medicine/European Society for Radiotherapy and Oncology/European Society of Urogenital Radiology/International Society of Urological Pathology/International Society of Geriatric Oncology (EAU/EANM/ESTRO/ESUR/ISUP/SIOG)— *Guidelines on Prostate Cancer*	Guidelines for MRI in biopsy decision *Recommendations in biopsy-naïve patients*	
	Perform MRI before prostate biopsy.	Strong
	When MRI is positive (i.e., PI-RADS ≥ 3), combine targeted and systematic biopsy	Strong
	*Recommendations in patients with prior negative biopsy*	
	Perform MRI before prostate biopsy	Strong
	When MRI is positive (i.e., PI-RADS ≥ 3), perform targeted biopsy only.	Weak
	Guidelines for first-line treatment of various disease stages *Active Surveillance*	
	Perform MRI before a confirmatory biopsy if no MRI has been performed before the initial biopsy	Strong
	Take both targeted biopsy (of any PI-RADS ≥ 3 lesion) and systematic biopsy if a confirmatory biopsy is performed.	Weak
	If a patient has had upfront MRI followed by systematic and targeted biopsies there is no need for confirmatory biopsies	Weak
NCCN—*Prostate Cancer Early Detection*	*Further evaluation and indications for biopsy*	
	mpMRI if available High suspicion for clinically significant cancer: Image-guided biopsy via TR or TP approach with MRI targeting (preferred) or without MRI targeting	It is strongly recommended that image-guided biopsy techniques be employed routinely
	*Management of biopsy results*	
	Atypical intraductal proliferation (AIP) without invasive carcinoma Repeat biopsy using MRI targeting and systematic biopsy to look for invasive carcinoma	AIP is potentially considered a marker of unsampled cancer, and it is associated with an increased risk (50%) of invasive carcinoma and/or intraductal carcinoma on repeat biopsy

**Table 2:** List of some current platforms for MRI–ultrasound fusion biopsy.

Vendor/Device	Ultrasound	Tracking Mechanism	Biopsy Route	FDA 510(k)
Invivo(Philips) UroNav	Manual sweep	Electromagnetic	Transrectal, ṗ transperineal	2005
Eigen Artemis	Manual rotation	Articulated arm	Transrectal	2008
Koelis Urostation	Automatic rotation	Image-based	Transrectal	2010
Pi Medical BiopSee	Biplane probe on stepper	Stepper with encoders	Transperineal	N/A
Esaote Virtual Navigator	Manual sweep/rotation	Electromagnetic	Transrectal	2014
BK Ultrasound BioJet Fusion	Biplane probe on stepper	Stepper with encoders	Transrectal or transperineal	2012
Hitachi/Real-Time Virtual Sonography	Real-time biplanar	Electromagnetic	Transrectal or transperineal	2010
MIM Software Symphony Bx	Biplane probe on stepper	Stepper with encoders	Transperineal	2014
Focal Healthcare Fusion Bx	Manual rotation	Articulated arm	Transrectal	2016
UC-Care Navigo	Manual sweep	Electromagnetic	Transrectal	2016

## Data Availability

Data from case description is unavailable due to privacy or ethical restrictions.
